# *Amomum nilgiricum* (Zingiberaceae), a new species from Western Ghats, India

**DOI:** 10.3897/phytokeys.8.2152

**Published:** 2012-01-06

**Authors:** V. P. Thomas, M. Sabu, K.M. Prabhu Kumar

**Affiliations:** 1 Department of Botany, University of Calicut, P. O. Calicut University 673 635, Kerala, India

**Keywords:** *Amomum*, Zingiberaceae, Western Ghats, Kerala, India

## Abstract

A new species of *Amomum* Roxb. from Western Ghats of Kerala is illustrated and described. *Amomum nilgiricum* VP.Thomas & M.Sabu, **sp. nov.** shows similarity with *Amomum masticatorium* Thwaites in having long drying ligule with an acuminate apex, pubescent anther and echinate capsules, but differs in clump forming habit with non-stoloniferous rhizomes, tomentose lamina beneath, long corolla tube, obovate to rhomboid labellum with clefted apex and without any colour design, emarginate anther crest and reduced staminodes. Detailed description, illustration, photographs, conservation status, and distributional details are provided.

## Introduction

Intensive botanical explorations for the revision of Indian *Amomum* have resulted in the collection of an interesting species with long membranous ligule from the Silent Valley National Park on the Western Ghats of Kerala. The family Zingiberaceae (ginger family) consists of 53 genera and over 1200 species (Kress et al. 2002). *Amomum* Roxb. is the second largest genus after *Alpinia* Roxb. within Zingiberaceae with about 150-180 species, widely distributed in Southeast Asia (Xia et al. 2004). In India the genus is represented by 22 species, mostly restricted to North-East India and South India (Thomas et al. 2010). Sabu (2006) reported 6 species of *Amomum* from South India and Thomas et al. (2009) raised the number species to 7 by reporting new distribution record of *Amomum fulviceps* Thwaites.

The new species, *Amomum nilgiricum*, shows similarity with *Amomum masticatorium* Thwaites in having long drying ligule with an acuminate apex, pubescent anther and echinate capsules, but differs in clump forming habit with non-stoloniferous rhizomes, tomentose lamina beneath, long corolla tube, obovate to rhomboid labellum with clefted apex, emarginate anther crest and reduced staminodes (Table 1). *Amomum nilgiricum* shows some morphological affinities with *Amomum villosum* group in the phylogenetic grouping of Xia et al. (2004).

**Table 1.** Distinguishing morphological characters of *Amomum masticatorium* and *Amomum nilgiricum*

**Attributes**	***Amomum masticatorium***	***Amomum nilgiricum***
Habit	slender, spreading	robust, clump forming
Rhizome	slender and stoloniferous	stout and non-stoloniferous
Lamina	oblong-lanceolate, 15−30 × 3−7.5 cm	lanceolate to elliptic-lanceolate, 32−41 × 6.5−8 cm
Petiole	0−2 mm long	2−8 mm long
Leaves	glabrous to puberulous beneath	tomentose beneath
Ligule	2.5−4.5 cm long and half deciduous	4.5−9 cm long and persistent
Corolla tube	shorter than labellum	longer than labellum
Labellum	3−3.5 × 2.3−2.8 cm, trilobed, maroon stripes on yellow ground	1.4−1.5 × 1−1.2 cm, not trilobed, uniformly yellow
Lateral staminodes	2−5 mm long	absent
Stamen	1.7−2.1 cm long, crest truncate, 1.5−1.6 × 0.3−0.4 cm	1.1−1.2 cm long, crest emarginate, *c*. 0.3 × 0.1 cm

## Taxonomic affinities

### Amomum nilgiricum

V.P. Thomas & M. Sabu sp. nov.

urn:lsid:ipni.org:names:77116671-1

http://species-id.net/wiki/Amomum_nilgiricum

Figs 1 2 

#### Diagnosis.

The species shows similarity with *Amomum masticatorium* Thwaites in having long drying ligule with an acuminate apex, pubescent anther and echinate capsules, but differs in clump forming habit with non-stoloniferous rhizomes, tomentose lamina beneath, long corolla tube, obovate to rhomboid labellum with clefted apex and without any colour design, emarginate anther crest and reduced staminodes.

#### Type.

**INDIA.** Kerala: Palakkad District, Silent Valley National Park, 1.5 km from Walakkad towards Sispara, 1200 m elevation, 3 April 2009, *V.P. Thomas & M.C. Shameer 115574* (holotype: CALI; Isotype, MH, CAL).

#### Description.

Clump forming herb. Rhizome non-stoloniferous, stout, robust, 2−4 cm thick, robust, creamy-white inside, sheathed with scales; scales ovate to triangular, chartaceous, c. 1.8 × 2 cm, apex nearly rounded, pubescent externally. Leafy shoots 200−400 cm tall, robust, clump forming; sheath 2.5−4.5 cm wide at base, green, densely pubescent externally. Leaves 14−20 per leafy shoot; lamina lanceolate to elliptic-lanceolate, 32−41 × 6.5−8 cm, base cuneate, margin slightly straight, apex acuminate to 3 cm long, puberulous to glabrous and green on upper surface, tomentose and pale beneath; midrib hispid beneath; veins appressed above; petiole 2−8 mm long, pale green, wooly tomentose. Ligule entire, lanceolate, 4.5−9 cm long, chartaceous, drying, persistent, apex acute, pubescent to tomentose externally, glabrous within. Inflorescence 7−15 cm long, many flowered, arise from the rhizome under soil; peduncle 3.5−7.5 cm long. Bract oblong, 3−4.7 × 1.6−2.1 cm, coriaceous, red, margin ciliate, apex slightly emarginate, pubescent externally, glabrous internally. Bracteole tubular, 2-lobed, 2.2−2.5 × 0.5−0.6 cm, unequally split, membranous, red, margin ciliate, apex acute, pubescent externally, glabrous within. Flower 4.7−5.2 cm long, yellow; pedicel 5 mm long. Calyx 2 or 3-lobed, 2.4−2.8 × 0.4 cm, pale red, membranous, split nearly equal, margin ciliate, apex acute, pubescent externally, glabrous within. Corolla tube 2.5−3 cm long, c. 4 mm wide at mouth, pale yellow, pubescent externally, glabrous internally except near mouth; dorsal corolla lobe oblong, 1.4−1.6 × 0.7−0.8 cm, yellow, margin ciliate, apex hooded, ecuspidate, pubescent externally, glabrous within; lateral corolla lobes oblong, 1.4−1.6 × 04−0.6 cm, yellow, margin ciliate, apex nearly rounded, one side slightly folded, pubescent outside, glabrous within. Labellum obovate to rhomboid, 1.4−1.5 × 1−1.2 cm, uniform yellow, margin entire, apex clefted, pubescent inside along the median part. Lateral staminodes absent. Stamen 1.1−1.2 cm long; filament 4−5 × 2.5−3 mm, pale yellow, broader towards base, rarely minutely pubescent; connective rarely pubescent externally; crest inconspicuous, c. 3 × 1 mm, yellow, apex emarginate, rarely puberulous; anther thecae oblong, 6−7 mm long, creamy-white, base nearly rounded, apex rounded, pubescent; dehiscing throughout their length. Epigynous glands 2, oblong, 3−4 mm long, cream coloured, apex truncate, rarely puberulous. Ovary globose, 4−5 × 4 mm, densely pubescent externally; locules 3; ovules many on axile placentae; style 3.4−3.7 cm long, pubescent towards tip, glabrous towards base; stigma, tubular, c. 1 mm across, pale yellow, mouth ciliate, opening terminal. Capsule 8−10 per spike, globose, 2−3 × 2−3 cm, red, echinate, spines stout, pubescent externally, calyx not persistent. Seeds many, slightly oblong, 4−5 × c. 3 mm, black, aromatic, arillate, glabrous; aril white.

**Figure 1.**
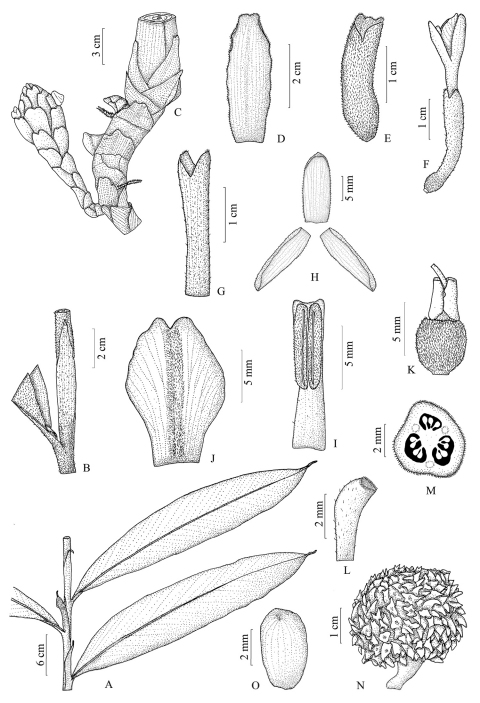
*Amomum nilgiricum* **A** a part of leafy shoot **B** ligule **C** inflorescence **D** bract **E** bracteole **F** flower **G** calyx **H** corolla lobes **I** stamen **J** labellum **K** ovary with epigynous glands and style **L** stigma **M** c.s. of ovary **N** fruit **O** seed.

**Figure 2.**
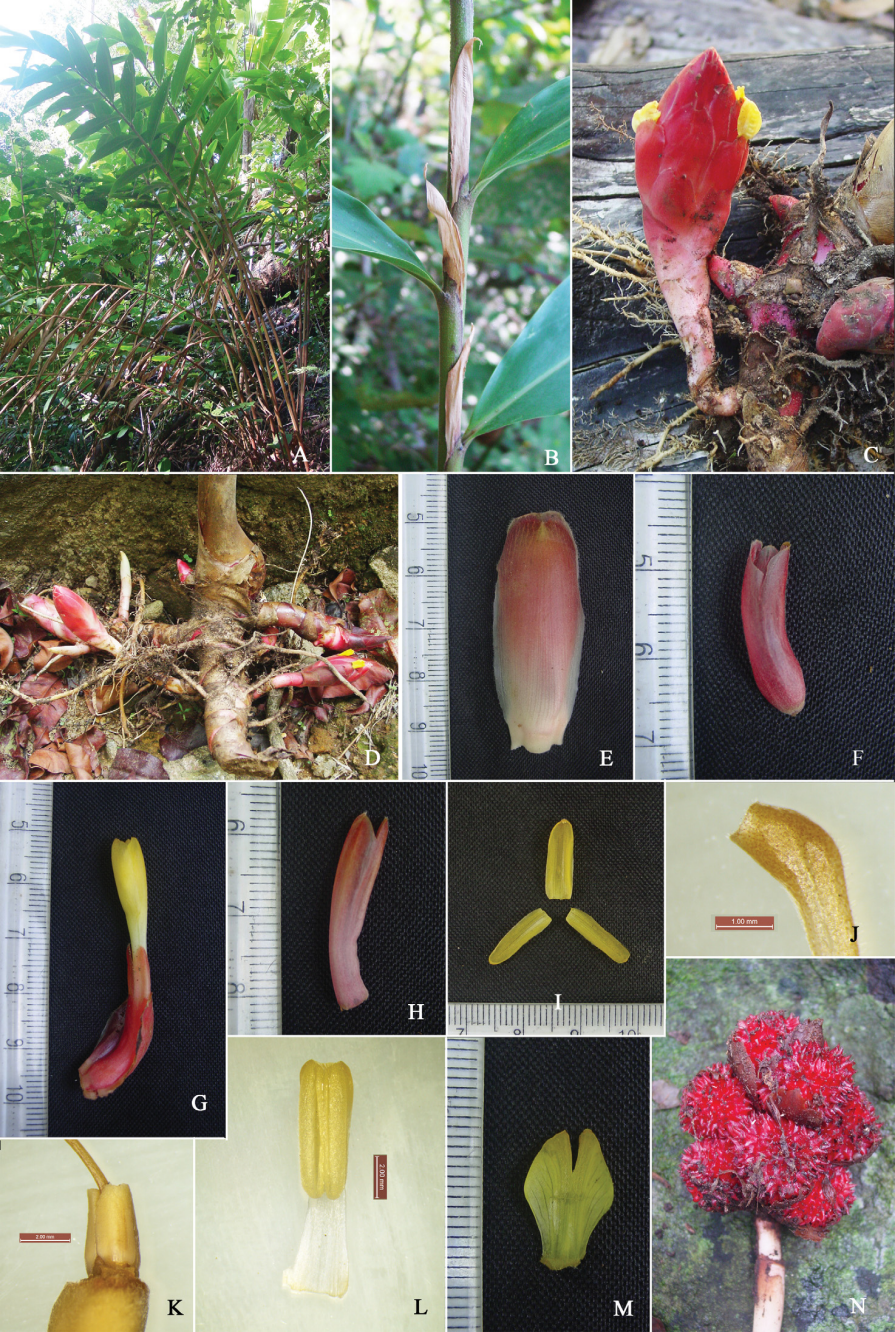
*Amomum nilgiricum* **A** habit **B** ligules **C** inflorescence **D** rhizome with inflorescences **E** bract **F** bracteole **G** flower with a bract **H** calyx **I** corolla lobes **J** stigma **K** ovary with epigynous glands and style **L** stamen **M** labellum **N** infructescence.

#### Flowering and fruiting.

March−November.

#### Distribution.

Known only from type locality, Silent Valley National Park, Western Ghats, Kerala in the evergreen forest above 1210 m.

#### Etymology.

the specific epithet *nilgiricum* indicates the place of collection Nilgiri Hills, a part of Western Ghats.

#### Conservation status.

Critically endangered (CR B1ab(ii,iii)+B2ab(i,ii)). The taxon has been evaluated against the criteria as described in IUCN (2001). The area of occupancy is estimated to be less than 10 Km^2^ and its habitat is severely fragmented, and known to exist only in a single location. A continuous decline in quality of habitat and extent of occurrence is noticed. Major threat to the population are forest fire and clearing of trekking path in the forest which cause damage to the existing population.

#### Specimens examined.

INDIA, Kerala: Palakkad District, Silent Valley National Park, 3 km from Walakkad towards Sispara, 24 September 2008, *V.P. Thomas & K.M. Prabhu Kumar 115504* (CALI); 2 km from Walakkad towards Sispara, 1 March 2009, *V.P. Thomas & A.V. Prasanth 115540* (CALI).

## Supplementary Material

XML Treatment for Amomum nilgiricum
